# Supplementary figures and images for: Comparative proteomics reveals mechanisms that underlie insecticide resistance in Culex pipiens pallens Coquillett
Source: PLoS Negl Trop Dis. 2021 Mar 25;15(3):e0009237. doi: 10.1371/journal.pntd.0009237 (PMC7993597; doi:10.1371/journal.pntd.0009237)

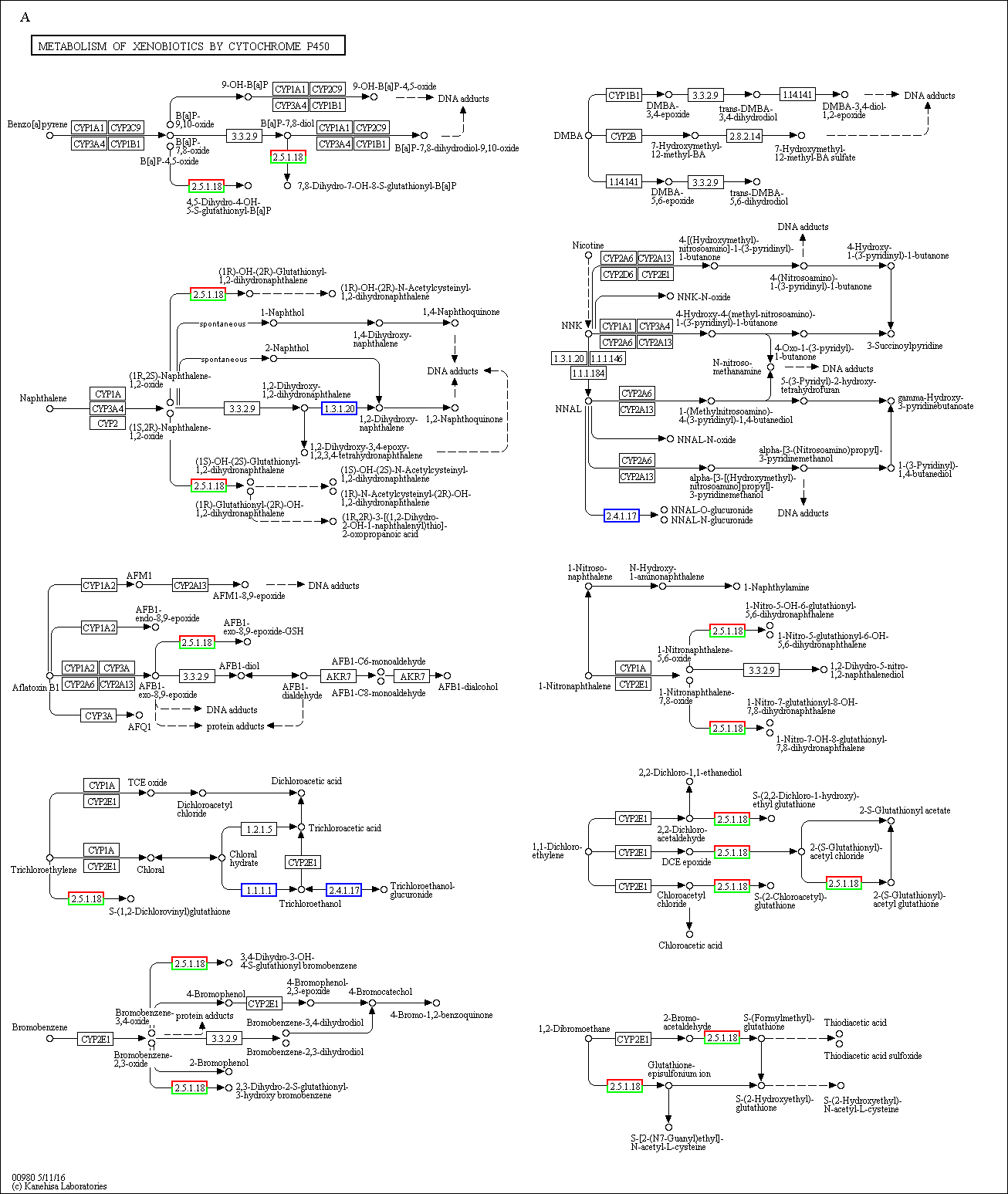

Supplement: S1 Fig — A. Representative KEGG metabolism of xenobiotics by cytochrome P450 pathway (map00980); B. Representative KEGG drug metabolism—cytochrome P450 pathway (map00982); C. Annotated KEGG map for Oxidative phosphorylation, map00190; D. Annotated KEGG map for Ribosome, map03010 The rectangular nodes in the figure represent gene products, the blue border belongs to the background proteins, and the white color indicates proteins not identified in this experiment. The red/green colors in the figure indicate to the differentially expressed proteins detected in this study, with red representing upregulated proteins and green downregulated proteins. Half red and half green indicates both upregulated and downregulated proteins for that gene product (the same meaning as in this manuscript). (TIF) [file pntd.0009237.s001.tif]

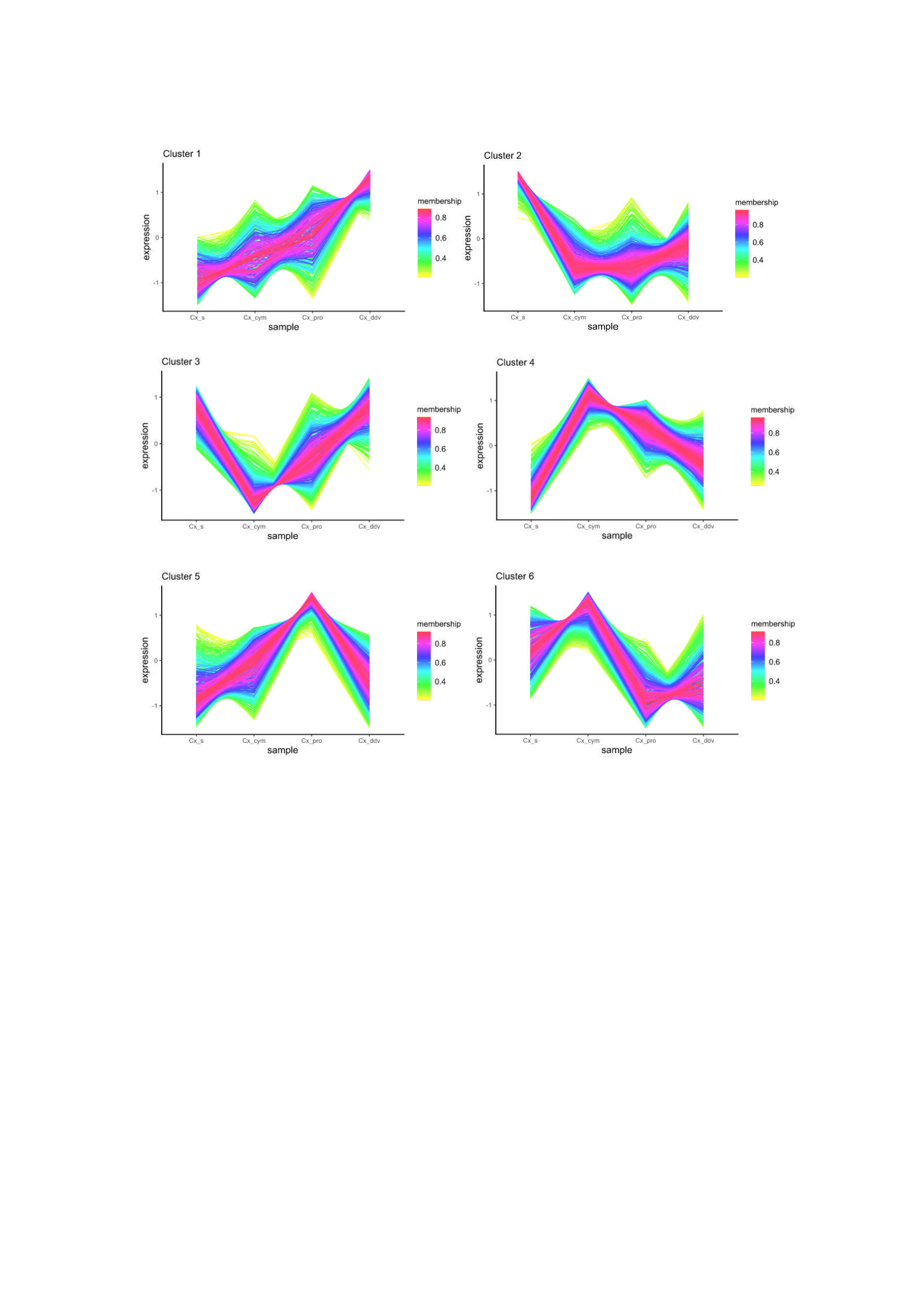

Supplement: S2 Fig — Each line in the figure represents a protein, and the different color representations show the relationship between relative expression and the mean value. Each graph shows one type of expression pattern, a trend that reflects changes in the expression of this group of proteins. (TIF) [file pntd.0009237.s002.tif]

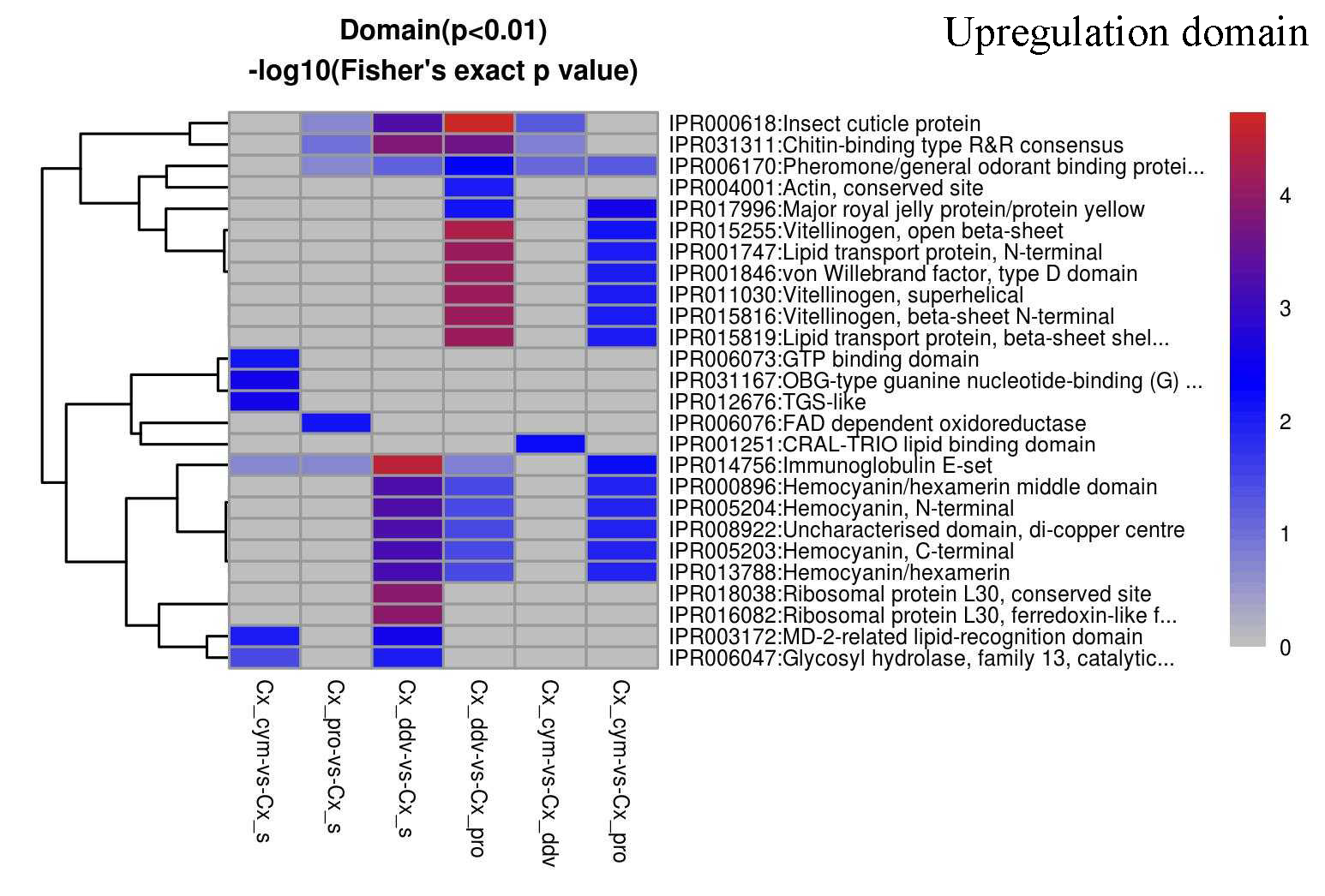

Supplement: S3 Fig — Clustering of the results of differential protein enrichment in different groups, the color −log10 (Fisher’s exact test p value) represents the credibility of enrichment. (TIF) [file pntd.0009237.s003.tif]

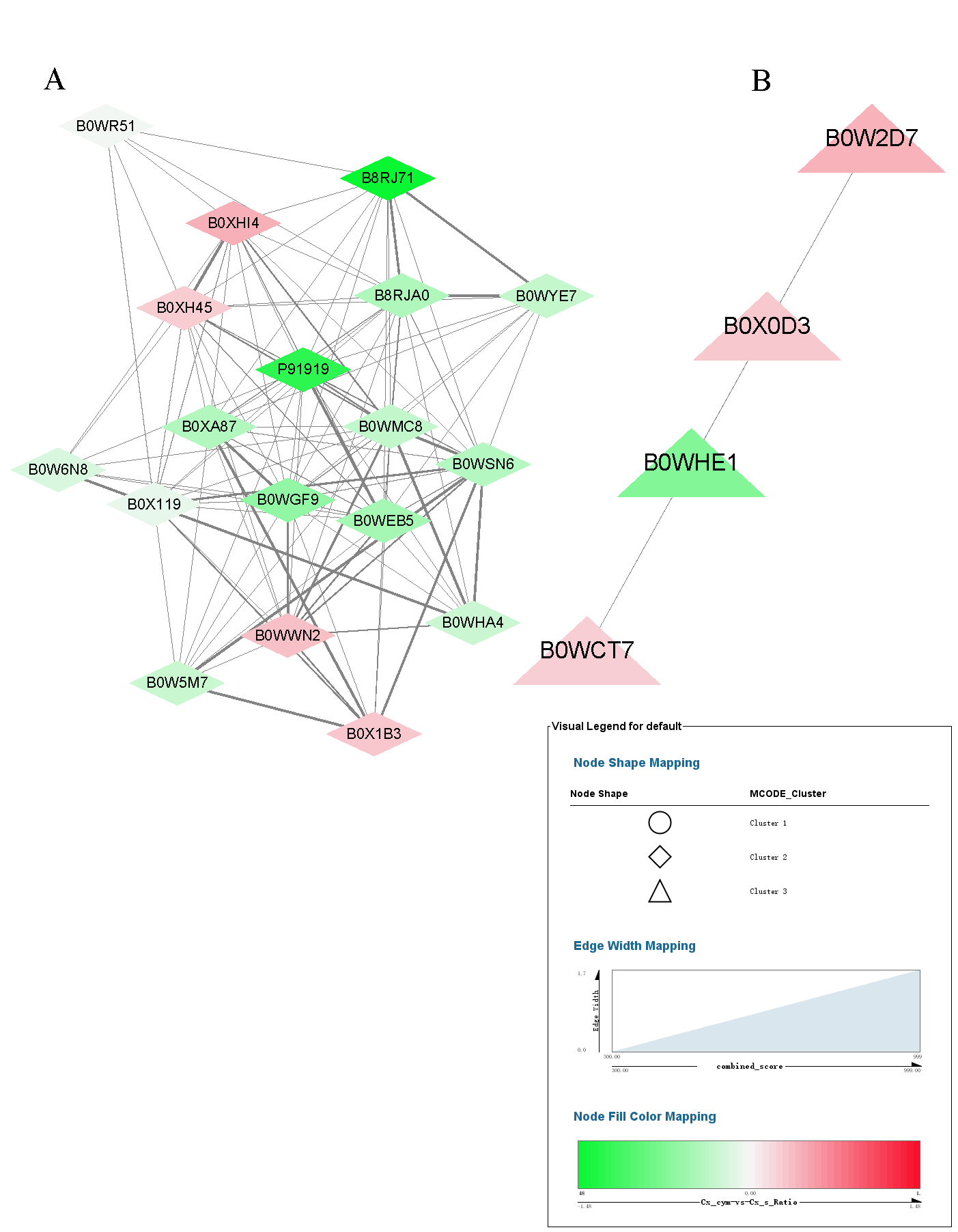

Supplement: S4 Fig — Network interaction analysis was performed using the STRING protein interaction prediction online software and was visualized using Cytoscape version 3.4 (TIF) [file pntd.0009237.s004.tif]

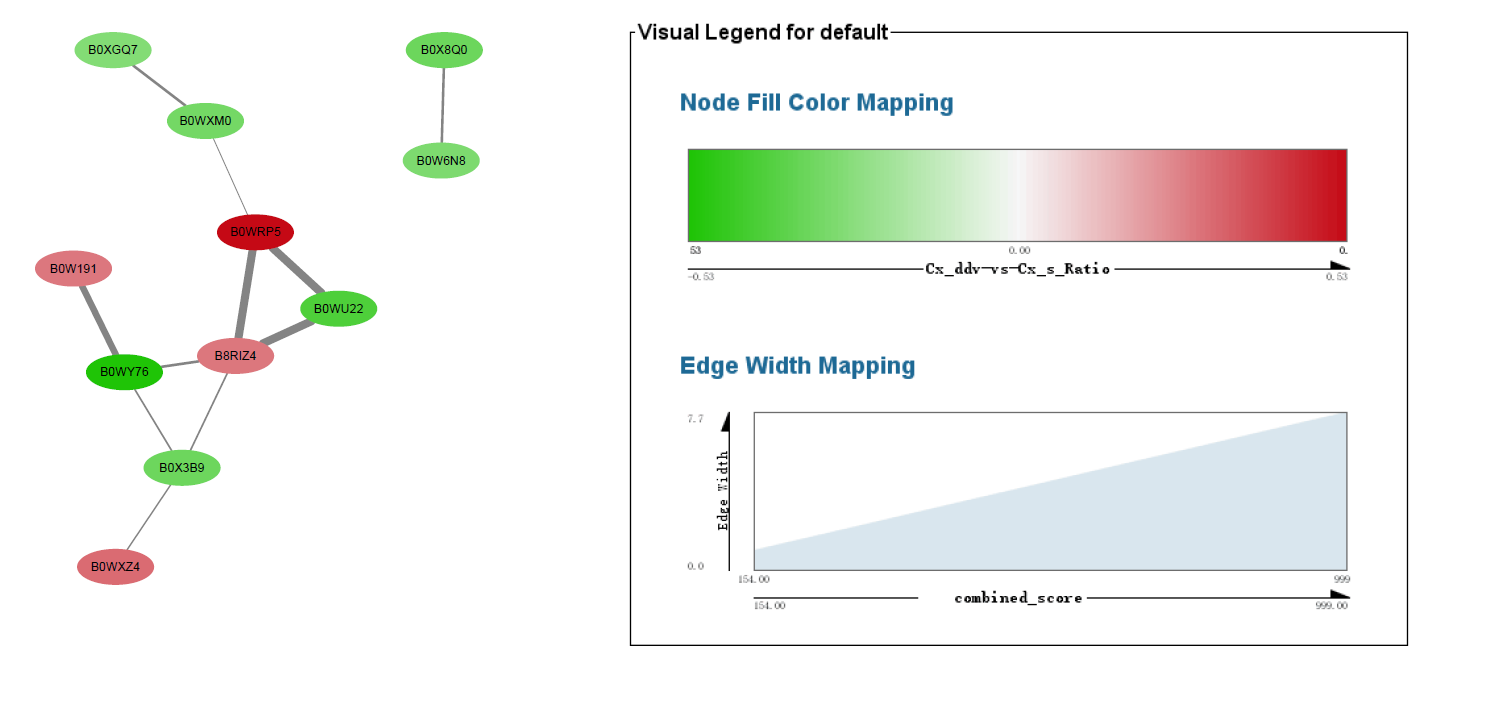

Supplement: S5 Fig — (TIF) [file pntd.0009237.s005.tif]

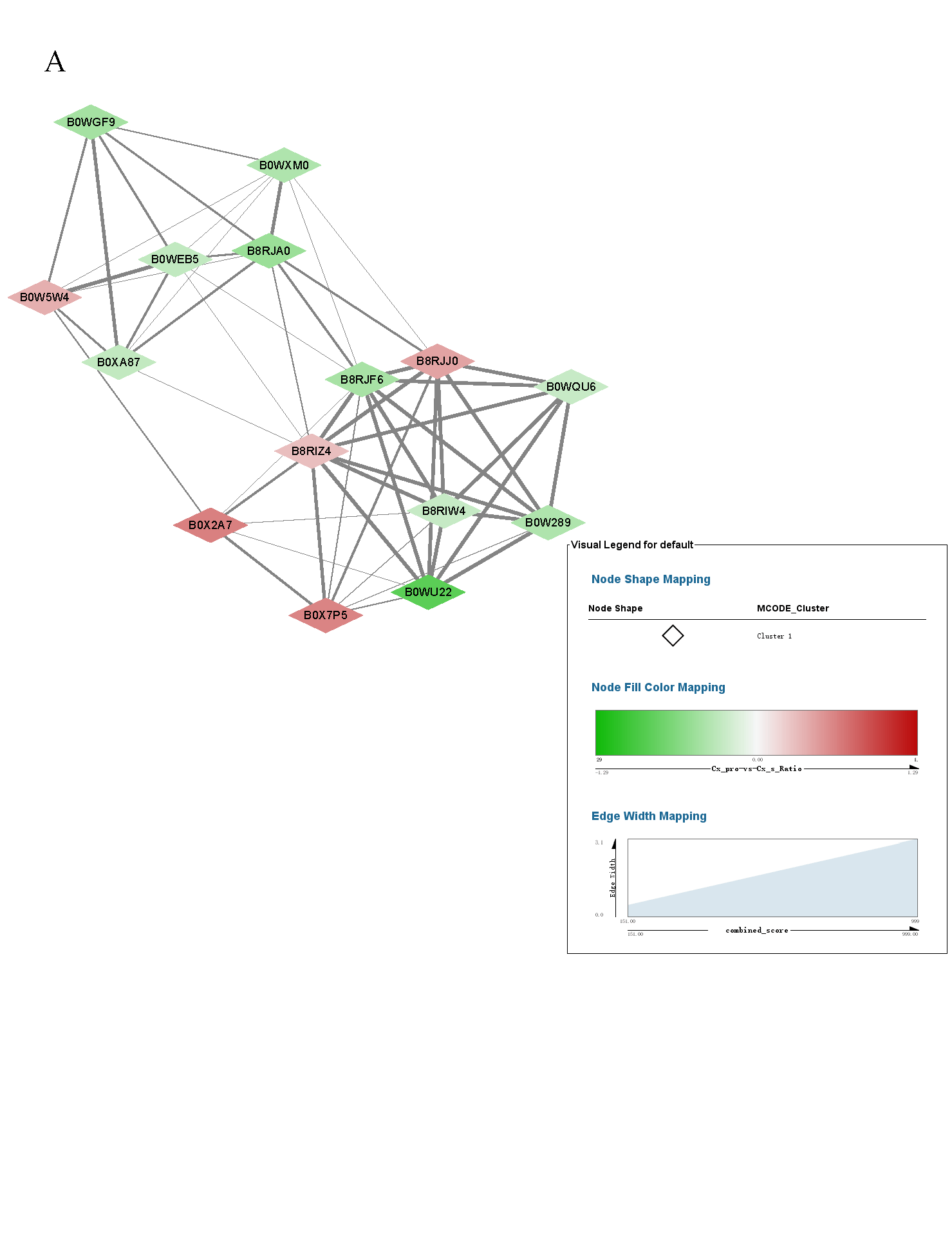

Supplement: S6 Fig — (TIF) [file pntd.0009237.s006.tif]

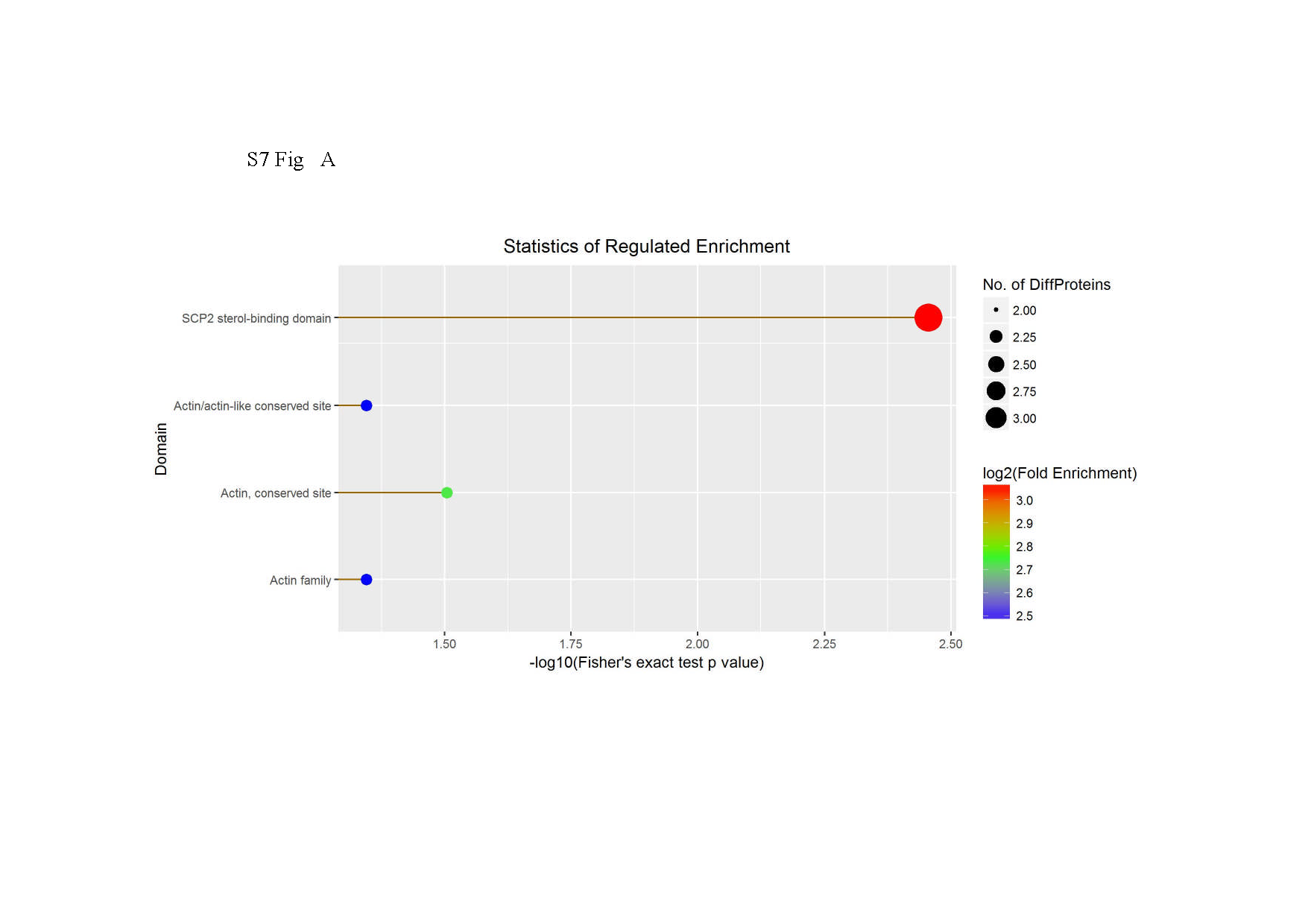

Supplement: S7 Fig — A, Significantly enriched domain in Cx_bti vs. Cx_nbti, B, Visualization of significantly enriched downregulation domain in Cx_bti vs. Cx_nbti. Proteins in functional categorizations of proteins differentially expressed according to Fisher’s extract test. The Number of Diffproteins is the number of differentially expressed proteins enriched in the Domain; the Domain enriched fold is shown on a log2 scale in a color gradient. (TIF) [file pntd.0009237.s007.tif]
