# Supplementary material for: Comparative proteomics reveals mechanisms that underlie insecticide resistance in Culex pipiens pallens Coquillett
Source: PLoS Negl Trop Dis. 2021 Mar 25;15(3):e0009237. doi: 10.1371/journal.pntd.0009237 (PMC7993597; doi:10.1371/journal.pntd.0009237)
Supplement: Attachment — Submitted filename: renamed_b0c63.docx [file pntd.0009237.s014.docx]

PLOS Neglected Tropical Diseases. Manuscript Number: PNTD-D-20-01279

*Culex pipiens pallens Coquillett* has multi-level molecular resistance mechanism adaptations to different insecticides by comparative proteomics analysis.

The authors discussed other mechanisms of insecticide resistance that are far from being fully understood, in addition to resistance at the target site. The work is important in that mosquito control based on chemical insecticides is considered an important element in current global strategies to control mosquito-borne diseases. The authors used a comparative proteomics approach. They have achieved excellent results. I recommend acceptance with minor revision.

But there is still room for improvement in the manuscript.

I have some observations to make:

Line 31: **Methods**

The authors did not indicate which part of the mosquito's body made up the sample. What solution was used to preserve the samples? How were the proteins extracted?

Line 40: correct “of which 1513”

Line 41: Why do you choose to work on midgut proteins? Are they more involved in the resistance?

Line 44: Authors should implicitly give the metabolic pathways involved in resistance.

Line 98-100: “………...large-scale expression profiling and screening for resistance-related proteins in mosquitoes are limited, two-dimensional gel electrophoresis (2-DE) proteomics [32,33], and iTRAQ” rephrase this sentence.

Line 102: “…...isobaric tags for relative and absolute quantification (iTRAQ)…...” Authors should give this full definition at the first mention in the text.

Line 114: **Methods**

I propose to make a simplified diagram that describes the different steps of the methods. This is to give an overall view of the activities.

Line 149: **Extraction of protein and proteolysis**

I propose to split into:

a-Protein extraction

b-Quantification of proteins

c-Trypsin digestion

d-Samples desalting

Line 259:” …………. 1513 protein obtained quantitative message………………….”

What does quantitative protein mean? Quantity relates to what?

Line 264: “………there were 164 proteins with significant differential expression (fold change >…...including 54 up-regulated and 110 down-regulated proteins.” Authors must color these lines in the (Supplementary table S1).

Line 295-296: “……………is activated associated with electron-transport chain subunits……………..” rephrase this sentence.

- The legend of the figures can be better improved. Authors should make a detailed description of the colours and meanings of clusters.
- Under no circumstances do I see any comparison between the different samplings (… 5 egg rafts, 5 fourth-instar larvae, 5 pupae, 2–3 days old 5 adult males, 5 adult females (without blood feeding) and midgut pools). What is the state of the proteins at larval and adult age?
- The document must be proofread by a native English speaker

e.g: mosquotes
