# Supplementary material for: Comparative proteomics reveals mechanisms that underlie insecticide resistance in Culex pipiens pallens Coquillett
Source: PLoS Negl Trop Dis. 2021 Mar 25;15(3):e0009237. doi: 10.1371/journal.pntd.0009237 (PMC7993597; doi:10.1371/journal.pntd.0009237)
Supplement: Attachment — Submitted filename: PLOS Neglected Tropical Diseases Reviewer 1 comments.doc [file pntd.0009237.s015.doc]

An interesting work, it was a pleasure reading. The author used proteome technology to study insecticides-associated resistance mechanism of *Culex pipiens pallens* and obtained some valuable data, which is helpful to clarify the specific ways and mechanisms of insecticide resistance in mosquitoes. I consider this study has important reference significance for the management of resistance to mosquitoes by using insecticides, and this paper is a valuable contribution to its field.

Below is a list of some comments and changes that might be considered:

1. Title - The title should be made some modification, this manuscript studied the insecticides-associated resistance mechanism by comparative proteomics, is not adaptations to different insecticides, so, I suggest the title revise to ‘Comparative proteomics reveals complex insecticides-associated resistance mechanism of Culex pipiens pallens Coquillett’ is much better.

2. Abstract - The abstract is concise and well documented. I don't have any comments about the same.

3. For KEGG pathways annotation, oxidative phosphophrylation (map00190), ribosome (03010), do you acquire KEGG Copyright Permission? If not, please provide.

4. One of key concerns for the manuscript is that there are some grammatical errors and English improvement need to be carried by professional editor. Some errors but not all that I found are listed below, and I guess there are more mistakes in this manuscript.

Line 74 ‘resulted in’ can change to ‘result in’

Line 250 ‘increased’ can change to ‘increase’

5. Results - Line 257, A total of 2,502 protein were identified in two replicates of the LC-MS/MS experiments with a high confidence of peptide selection (FDR=0.01). Among them, 1513 protein obtained quantitative message, why were some proteins not quantified?

6. Material - Line 197: What UniProt dataset is used for the annotation? I do not believe a Culex pipiens pallens proteome annotation set exists at this point. Are you using the Culex quinquefasciatus annotation set? The Drosophila melanogaster set? Or something else?

7. Line 214-217: Same question as above, Culex pipiens pallens is not an available species for KEGG mapping, what dataset is being used?

8. Line 281: What program/algorithm was used to generate the hierarchical clustering? What settings were used?

9. Line 328-332: Again, Culex pipiens pallens is not included in the STRING database, what protein network is used to predict the identified protein interactions? What settings were used for this analysis?

10. Figure 3: What do the blue highlighted proteins represent? The caption states that “differentially expressed proteins were mapped as red and green”. Is red more abundant and green less abundant? What does it mean when a protein is mapped in BOTH red and green as in figure 3?

11. Figure 4: What is the y-axis scale in these plots – Log2(fold change), raw fold change?

12. Figure S1: Annotated KEGG map for oxidative phosphorylation, map00190, the caption states “The enzymes marked by the red frame is related to up-regulated gene, and the enzyme labeled by the green frame is related to down-regulated gene.” This raises several questions. First, is this showing gene expression data rather than protein abundance data? If so, where is this data coming from and how does it relate to the proteomics experiment described in the manuscript? Many enzymes are marked in blue, what does this color represent?

13. Figure S5: What does a figure contribute? It appears to show the entire COG Function Classification map with none of the measured protein data included?

I would recommend that this manuscript could be accepted for publication after minor revisions.
