# Supplementary material for: Comparative proteomics reveals mechanisms that underlie insecticide resistance in Culex pipiens pallens Coquillett
Source: PLoS Negl Trop Dis. 2021 Mar 25;15(3):e0009237. doi: 10.1371/journal.pntd.0009237 (PMC7993597; doi:10.1371/journal.pntd.0009237)
Supplement: Attachment — Submitted filename: PLOS NTD Reviewer 1-3 Point rebuttal.doc [file pntd.0009237.s016.doc]

**Reviewer 1**

1. Title - The title should be made some modification, this manuscript studied the insecticides-associated resistance mechanism by comparative proteomics, is not adaptations to different insecticides, so, I suggest the title revise to ‘Comparative proteomics reveals complex insecticides-associated resistance mechanism of Culex pipiens pallens Coquillett’ is much better.

A: I agree with your opinion, so I made some change of the title.

2. Abstract - The abstract is concise and well documented. I don't have any comments about the same.

3. For KEGG pathways annotation, oxidative phosphophrylation (map00190), ribosome (03010), do you acquire KEGG Copyright Permission? If not, please provide.

A: KEGG Copyright Permission was uploaded as Additional file (KEGG Copyright Permission)

4. One of key concerns for the manuscript is that there are some grammatical errors and English improvement need to be carried by professional editor. Some errors but not all that I found are listed below, and I guess there are more mistakes in this manuscript.

Line 74 ‘resulted in’ can change to ‘result in’

Line 250 ‘increased’ can change to ‘increase’

A: AJE Editing Certificate was uploaded as Additional file.

5. Results - Line 257, A total of 2,502 protein were identified in two replicates of the LC-MS/MS experiments with a high confidence of peptide selection (FDR=0.01). Among them, 1513 protein obtained quantitative message, why were some proteins not quantified?

A: Some proteins not quantified because of these proteins are trace.

6. Material - Line 197: What UniProt dataset is used for the annotation? I do not believe a Culex pipiens pallens proteome annotation set exists at this point. Are you using the Culex quinquefasciatus annotation set? The Drosophila melanogaster set? Or something else?

A: The UniProt dataset used for the annotation is the *Culex quinquefasciatus* annotation set (https://www.uniprot.org/uniprot/?query=taxonomy:7174), get the ID of the protein based on the protein search，ID of the protein is converted to the ID of the UniProtKB database and then based on the ID of the UniProKB at UniProt to find the GO annotation information in Culex quinquefasciatus, was uploaded as Additional file (uniprot-taxonomy__Culex [7174]_.fasta.gz).

7. Line 214-217: Same question as above, *Culex pipiens pallens* is not an available species for KEGG mapping, what dataset is being used?

 A: The KEGG database used for the annotation with the species Culex quinquefasciatus.

8. Line 281: What program/algorithm was used to generate the hierarchical clustering? What settings were used?

A: Line 263-274, Expression-based and functional enrichment-based clustering for different protein groups was used to explore the potential relationships between different protein groups with special protein functions. A heatmap was generated by the software Heml 1.0.3. The expression pattern of the protein was completed by using the timeclust function in the R package ‘TCseq’. First, we collated all of the protein groups obtained after functional enrichment analysis along with their P values. Second, we sorted those categories enriched in at least one of the protein groups with a P value <0.05. This filtered P value matrix was transformed by the function x = −log10 (P value). Third, z-transformation was applied to x values for each functional category, and z scores were clustered by one-way hierarchical clustering (Euclidean distance, average linkage clustering). Finally, cluster membership was visualized by a heat map using the “pheatmap” function from the R package.

9. Line 328-332: Again, Culex pipiens pallens is not included in the STRING database, what protein network is used to predict the identified protein interactions? What settings were used for this analysis?

A: The STRING database with the species Culex quinquefasciatus. Select the combined score > 0.400 between nodes for PPI with Cytoscape, part of which shows in paper only containing important proteins.

10. Figure 3: What do the blue highlighted proteins represent? The caption states that “differentially expressed proteins were mapped as red and green”. Is red more abundant and green less abundant? What does it mean when a protein is mapped in BOTH red and green as in figure 3?

A: In figure legends the comments have already included, Figure 3. A: Representative KEGG metabolism of xenobiotics by cytochrome P450 pathway (map00980), B: Representative KEGG drug metabolism - cytochrome P450 pathway	(map00982), the rectangular nodes in the figure represent gene products. The blue border belongs to the background proteins, and the white colour indicates proteins not identified in this experiment. The red/green colours in the figure belong to the differentially expressed proteins detected in this study, in which red represents upregulated proteins and green represents downregulated proteins. Half red and half green indicates that both upregulated and downregulated proteins are present for that gene product.

11. Figure 4: What is the y-axis scale in these plots – Log2(fold change), raw fold change?

A: In figure legends, Comparison of protein expression pattern clusters between Cx_cym, Cx_pro, Cx_ddv and Cx_s in Cx. pipiens pallens. For the expression trend line graph of each sub-cluster, the x-axis is the comparison sample group, and the y-axis is the relative expression level of the protein in the group of samples. Each line in the figure represents a protein, and the different colour representations show the relationship between relative expression and the mean value. Each graph shows one type of expression pattern, a trend that reflects changes in the expression of this group of proteins.

12. Figure S1: Annotated KEGG map for oxidative phosphorylation, map00190, the caption states “The enzymes marked by the red frame is related to up-regulated gene, and the enzyme labeled by the green frame is related to down-regulated gene.” This raises several questions. First, is this showing gene expression data rather than protein abundance data? If so, where is this data coming from and how does it relate to the proteomics experiment described in the manuscript? Many enzymes are marked in blue, what does this color represent?

A: In figure legends, the blue border belongs to the background protein, and the black border indicates the protein was not identified in this experiment. The red/green colour in the figure marks the differentially expressed proteins detected in this experiment, in which red represents upregulated proteins and green represents downregulated proteins. Half red and half green indicates the protein is both upregulated and downregulated.

13. Figure S5: What does a figure contribute? It appears to show the entire COG Function Classification map with none of the measured protein data included?

A: In figure legends, 559 proteins were assigned to 15 COG categories, and the “J” cluster (translation, ribosomal structure and biogenesis) and “Z” cluster (cytoskeleton ) represent the largest two groups.

**Reviewer 2**

Line 31: **Methods**

The authors did not indicate which part of the mosquito's body made up the sample. What solution was used to preserve the samples? How were the proteins extracted?

A: Line 181-184, Each sample (the Cx_cym, Cx_pro, Cx_ddv and Cx_s strains) was extracted separately from 5 egg rafts, 5 fourth-instar larvae, 5 pupae, five 2- to 3-day-old adult males, 5 adult females (without blood feeding) and midgut pools of Cx. pipiens pallens. For the egg, larvae, pupae, adult males and females, the whole mosquitoes’ body made up the sample.

For preserve the samples, all the mosquitoes were stored at −80 ℃.

The proteins extracted, see line 180.

Line 40: correct “of which 1513”

A: AJE Editing Certificate was uploaded as Additional file.

Line 41: Why do you choose to work on midgut proteins? Are they more involved in the resistance?

A: There are digestive tract linings and metabolic enzymes in the midgut of mosquitoes, so, Cx. pipiens pallens mosquitoes were selected at the larval stage, larvae of mosquitoes drink and eat water mix with the Bacillus thuringiensis subspecies israelensis (bti), midgut of Cx. pipiens pallens mosquitoes exposed with bti directly, different protein expression in the midgut of Cx. pipiens pallens were valided.

Line 44: Authors should implicitly give the metabolic pathways involved in resistance.

A: metabolic pathways involved in resistance were added.

Line 98-100: “………...large-scale expression profiling and screening for resistance-related proteins in mosquitoes are limited, two-dimensional gel electrophoresis (2-DE) proteomics [32,33], and iTRAQ” rephrase this sentence.

A: AJE Editing Certificate was uploaded as Additional file.

Line 102: “…...isobaric tags for relative and absolute quantification (iTRAQ)…...” Authors should give this full definition at the first mention in the text.

A: Line 35, Yes, I have already changed, and give this full definition at the first mention in the text

Line 114: **Methods**

I propose to make a simplified diagram that describes the different steps of the methods. This is to give an overall view of the activities.

Line 149: **Extraction of protein and proteolysis**

I propose to split into:

a - Protein extraction

b - Quantification of proteins

c - Trypsin digestion

d - Samples desalting

A: Yes, I have made some modifications.

Line 259:” …………. 1513 protein obtained quantitative message………………….”, what does quantitative protein mean? Quantity relates to what?

A: Quantitative protein means that these proteins were quantification, the amount of these proteins were more than trace protein.

Line 264: “………there were 164 proteins with significant differential expression (fold change >…...including 54 up-regulated and 110 down-regulated proteins.” Authors must color these lines in the (Supplementary table S1).

A: Line 306, Supplementary table S2 was uploaded as Additional file.

Line 295-296: “……………is activated associated with electron-transport chain subunits……………..” rephrase this sentence.

A: AJE Editing Certificate was uploaded as Additional file.

- The legend of the figures can be better improved. Authors should make a detailed description of the colours and meanings of clusters.

A: I have made some modifications.

- Under no circumstances do I see any comparison between the different samplings (… 5 egg rafts, 5 fourth-instar larvae, 5 pupae, 2–3 days old 5 adult males, 5 adult females (without blood feeding) and midgut pools). What is the state of the proteins at larval and adult age?

A: Line 191-196, Then, a total of 100 μg protein/sample was combined from the egg, larvae, pupae, and adult male and female stages (20 μg each), so, in the manuscript, we do not compare between the different samplings, we compare mix samples of Cx_cym, Cx_pro, Cx_ddv, and Cx_s strains.

Protein differential expression of Egg, larvae, pupae, adult males and females were compare in the next manuscript (Unpublished data).

- The document must be proofread by a native English speaker

e.g: mosquotes

A: We have already proofread by a native English speaker, AJE Editing Certificate was uploaded as Additional file.

**Reviewer #3:**

1. It can be better presented that a set of markers for future studies was obtained. Novel markers that can be further studied should be better (clearly) stressed. Maybe - section containing "Future perspectives" can be useful.

A: I think in the discussion have already including the future perspectives.

2. For clarity, I strongly recommend simplify and edit the KEGG presentations. Please remove unnecessary content. The paper will be then better readable and easier to understand. In the KEGGs there are pathways without any mark/connection – why?

A: In the KEGGs there are pathways without any mark/connection is that several specific proteins in this metabolic process were identified, other proteins that upstream and downstream relationship with these identified proteins are not identified (due to strict cutoff value, incomplete protein database of mosquitoes in the metabolic system, and so on).

3. It is recommended to mention some more relevant studies that investigated the insecticide resistance using proteomics. Authors reference “iTRAQ” study such as doi: 10.1186/s13071-015-0709-5, however, there are different technologies used, for the proteomic profiling and searching the markers. It would be interesting if the authors could stress more studies that investigated the resistance mechanisms in various insects using the high-throughput proteomics approach – in addition, the introduction could not be limited only to the mosquito. What is the difference (compared to proteomics) if the study is performed on transcript level? Or if the resistance is studied based on changes in the DNA sequence (aka the kdr resistance e.g. doi: 10.1371/journal.pone.0215541, https://doi.org/10.1155/2018/3098575)? Authors stress in the conclusion targeting the RNAi. They also stress cuticular, cytochrome P450s and glutathione S-transferase proteins. Indeed, the resistance (especially multiresistance adaptation) can also have the epigenetic basis, which is often overlooked. These mechanisms can be responsible for the multiple resistance mechanisms rather than kdr or the change in CYPs (that respond usually to the specific chemicals-pesticides). See for example DOI: 10.1016/j.jprot.2016.09.016. If you are able provide discussion on such markers (epigenetic), it can be important for future studies. Resistance may not be “only” kdr, detoxification enzymes (CYPs, GSTs, and other detoxification enzymes such as esterases/lipases, UGTs). As I can see from your results, the ribosomal proteins were importantly changed this appears also of importance, because of translation modulation can be --- linked beyond the ribosome. Further, there is also interesting the vitellinogen change (It may be of importance that there was observed change in vitellogenin isoforms due to deltamethrin treatment 10.1016/j.jprot.2016.09.016).

A: I have already stress more studies that investigated the resistance mechanisms in various insects using the high-throughput proteomics approach.

As for what is the difference (compared to proteomics) if the study is performed on transcript level? You can see article research on pre- and non-diapause locust eggs, an overall relatively low correlation between the expression levels of mRNAs and the abundance of various proteins has been reported in a comparison of prediapause and non-diapause locust eggs, expression profiles are highly dynamic, and mRNA expression and protein levels do not always correlate .

1. Tu X, Wang, Jie, Hao, Kun, Whitman, Douglas W., Fan, Yaoli, Cao, Guangchun, Zhang, Zehua (2015) Transcriptomic and proteomic analysis of pre-diapause and non-diapause eggs of migratory locust, Locusta migratoria L. (Orthoptera: Acridoidea). Scientific Reports 5.

2. Nie L WG, Zhang W (2006) Correlation between mRNA and protein abundance in desulfovibrio vulgaris: A multiple regression to identify sources of variations. Biochem Biophys Res Commun 339: 603–610.

3. Bolognani F P-BN (2008) RNA-protein interactions and control of mRNA stability in neurons. J Neurosci Res 86: 481–489.

In discussion, I add some discussion on the ribosomal proteins.

4. Further notes:

- Full names “Bacillus thuringiensis var. Israelensis“ are provided at lines 139 and 142 – please revise appropriate use of abbreviations throughout the text

A: I have revised appropriate use of abbreviations *Bacillus thuringiensis* subspecies *israelensis*.

- Line 156: here and elsewhere, please provide x g instead of rpm (rpm does not provide exact info about the centrifugal force because depending on the rotor size)

A: I have revised.

- Could you please provide the raw data of LC-MS/MS analyses performed?

- Could you please provide number of sequences and the date of downloading?

A: Data Accessibility Statement, Data can be found in Dryad Digital Repository (<https://doi.org/10.5061/dryad.9kd51c5fj>).

- Lines 209, 215 – again, you provide full names together with abbreviation “Kyoto Encyclopedia of Genes and Genomes (KEGG)“.

A: I have revised.

- Should “z scores“ should be, Z-score“ ?

A: I have revised.

- Line 236: proteine-protein - protein-protein -„ involved in cuticular resistence“ – what does it mean?

You mention the “cuticle”/”cellular cytoskeletal rearrangement“ …. These changes are really hard to explain… However, a proteomic study showed in heads of non-target Bombus terrestris important change in cuticle proteins (in particular cuticle protein 18.7-like; cuticle protein 21-like in Table 1 of https://doi.org/10.1016/j.jprot.2018.12.022) or the actin binding troponin C and filamin-A due to sublethal insecticide treatment. In that study it was suggested, these changes may reflect partial compensation for the disrupted synthetic pathways, such as the generation of membrane proteins.“ It is possible, that this suggestion may be useful to your story – especially in the discussion (Lines 407-417).

A: I have revised and add some in the discussion.

Maybe ??? Is it possible that the resistant insect to pesticides can be differently adapted to the stress linked to changes in the structural proteins?

Indeed, the response of targets as well as non-targets to pesticides appears complex. The tolerance/resistance mechanisms developed can be surprising - as indicated by various high-throughput studies. Unfortunately, some (such) important changes in markers are underestimated in most studies that focus primarily to the detoxification enzymes.

Be aware - the proteins presented as “cuticular/cuticle” the function and localization of the proteins should be confirmed, but most of the proteins are assigned based on similarity. This is important to consider also for number of other proteins. Overall, the changes of the high-throughput analysis give sense together – because some markers change because another markers also change….

A: I have revised and add some in the discussion.
