# Supplementary material for: Comparative proteomics reveals mechanisms that underlie insecticide resistance in Culex pipiens pallens Coquillett
Source: PLoS Negl Trop Dis. 2021 Mar 25;15(3):e0009237. doi: 10.1371/journal.pntd.0009237 (PMC7993597; doi:10.1371/journal.pntd.0009237)
Supplement: Attachment — Submitted filename: 2020.12.06 PLOS NTDs Reviewer Point rebuttal.doc [file pntd.0009237.s017.doc]

**Response letter how these points along with those from the reviewers**

1. Along with the specific comments from the reviewers, please ensure that the Dryad link is active.

A: Dryad link is active now. The file (Culex_P3_R2.msf) is too big (8.46 G), so it was put in another Dryad link <https://doi.org/10.5061/dryad.pzgmsbcjs>.

2. One of the reviewers commented that the replicate description is not adequate. Please directly indicate that biological replicates used for measuring protein levels. This needs clarification as three lines are mentioned for each selection, so if all lines were examined then at 24 proteomic samples are required to analyze the samples as described (only 8 are shown in table 1). If only a single line is examined twice, this needs to be mentioned as the results are less strong and this requires a correlation analysis between the replicates.

A: Fig 1 Correlations of differentially expressed proteins, relation between two biological replicates values

3. The figures need improvement as example, the labeling in Figure 5 and 6 overlap. In addition, it is very likely that figures can be combined.

A: I agree, so Fig 6 has been deleted.

4. The author revised the original manuscript with great effort. All concerns have been well addressed and I recommend acccepting this article for publication. However, the author needs to check the text and change the word "drug" with "insecticide", For example, a similar error occurs in Figure 3.b.

A: Line 53, 344, 590, 690, in the biological pathways it is the word drug, not insecticide.
