# Supplementary material for: Comparative proteomics reveals mechanisms that underlie insecticide resistance in Culex pipiens pallens Coquillett
Source: PLoS Negl Trop Dis. 2021 Mar 25;15(3):e0009237. doi: 10.1371/journal.pntd.0009237 (PMC7993597; doi:10.1371/journal.pntd.0009237)
Supplement: Attachment — Submitted filename: 2020.12.16 PLOS NTDs Reviewer rebuttal.doc [file pntd.0009237.s018.doc]

1. Labels need to be added to the scale for each figure.

A: I have already check and revised.

2. Parts of the figures are cut off.

A: I have already check and revised.

3. Labels are overlapping in Figure 6..

A: This is a clustering based on enrichment, which sums up the enriched items into "bold" ones

4. Format references.

A: I have already check and revised.

5. Please check the manuscript for grammar and format issues.

A: I have already check and revised.
