# Supplementary material for: Comparative proteomics reveals mechanisms that underlie insecticide resistance in Culex pipiens pallens Coquillett
Source: PLoS Negl Trop Dis. 2021 Mar 25;15(3):e0009237. doi: 10.1371/journal.pntd.0009237 (PMC7993597; doi:10.1371/journal.pntd.0009237)
Supplement: Attachment — Submitted filename: 2021.01.20 PLoS NTSs Review 4th.doc [file pntd.0009237.s019.doc]

Reviewers’ point rebuttal

1. Please correct the references by hand. There are many errors, specifically species names should be in italics and other aspects of the references aren't uniform.

A: I have already corrected these references.

2. The scale labels are still missing. There are not units. Are these fold change, z scores, etc?

A: There are not units in these figures, and these are fold change, Z-scores.

3. Figure 6: Revigo will output an editable pdf. Labels should not overlap. Also, what setting were used for this program.

A: I have already corrected.

4. All abbreviations need to be defined in the figure legends.

A: I have already corrected.

5. Some of the figure legends lack details. For example, the title does not necessarily reflect what is described in the legend. Please review all figure titles and legends.

A: I have already checked and made some modification.

6. What do the cluster represent in Fig. 4? This is not integrated well into the results.

A: Fig. 4 can not describe the insecticides-associated mechanism of *Culex pipiens pallens*, so I delete this figure.
