# Supplementary material for: Comparative proteomics reveals mechanisms that underlie insecticide resistance in Culex pipiens pallens Coquillett
Source: PLoS Negl Trop Dis. 2021 Mar 25;15(3):e0009237. doi: 10.1371/journal.pntd.0009237 (PMC7993597; doi:10.1371/journal.pntd.0009237)
Supplement: Attachment — Submitted filename: 2021.02.08 Review 5th.docx [file pntd.0009237.s020.docx]

Reviewers’ point rebuttal

1. There is still no indication that the scales for each figure are fold change, z score, etc. This still needs correction in all figures. As an example, there is no label on scale bar for Figure 1 and no indication what the scale represents in the legend. This needs correction as the reader would have no idea what this figure represents based on Figure 1 and the associated legend. Similarly, the scale labels should be moved next to the scale bar in Figure 2 and Figure 3.

A: I have already revised.

2. The figure legends still lack specific details to be read independently, such as abbreviations should be discussed, what is actually significantly upregulated, bold vs. normal font differences, and any other details. Also, please delete "Visualization of" as this is redundant.

A: I have already revised.

3. Fisher is not spelled correctly in Figure 5.

A: I have already corrected.

4. For clarity purposes, please move the Figure legends to the end of the document.

A: I have already corrected .
